# Telescopes

**Published:** 1892-11

**Authors:** 


### TELESCOPES.

The greatest refracting telescopes yet known are made by Alvin G. Clark, of Cambridgeport, Mass. So fine is the work required on the lenses of these instruments that the glassmakers commenced work on two disks, from which a forty-inch lens is to be made, four years ago, and only one has, as yet, been sent to Mr. Clark. If there is the most minute speck of any kind in the glass it is rejected. A disk forty inches in diameter and ten inches thick costs $8,000. After Mr. Clark has determined what curve to give the glass, an iron casting is made of the size and shape required. The disk is revolved upon this, and ground with steel crushings. Next, eight courses of emery and an adjustable tool are used, and at this stage measurements are made with an instrument that measures one-thirty-thousandth of an inch. The final shaping is made with beeswax and rouge, and even the bare thumb does its part in the polishing. The lens must be so exact in its curve that every ray striking it shall centre at a predetermined mathematical point.

